# Modified AS1411 Aptamer Suppresses Hepatocellular Carcinoma by Up-Regulating Galectin-14

**DOI:** 10.1371/journal.pone.0160822

**Published:** 2016-08-05

**Authors:** Yuri Cho, Yun Bin Lee, Jeong-Hoon Lee, Dong Hyeon Lee, Eun Ju Cho, Su Jong Yu, Yoon Jun Kim, Jong In Kim, Jong Hun Im, Jung Hwan Lee, Eun Ju Oh, Jung-Hwan Yoon

**Affiliations:** 1 Department of Internal Medicine and Liver Research Institute, Seoul National University College of Medicine, Seoul, Republic of Korea; 2 Department of Internal Medicine, CHA Gangnam Medical Center, CHA University, Seoul, Republic of Korea; 3 Department of Internal Medicine, CHA Bundang Medical Center, CHA University, Seongnam, Republic of Korea; 4 Aptamer Initiative, POSTECH Biotech Center, Pohang University of Science and Technology, Pohang, Republic of Korea; 5 Department of Internal Medicine, Seoul Metropolitan Government Seoul National 26 University Boramae Medical Center, Seoul, Republic of Korea; Yonsei University College of Medicine, REPUBLIC OF KOREA

## Abstract

Aptamers are small synthetic oligonucleotides that bind to target proteins with high specificity and affinity. AS1411 is an aptamer that binds to nucleolin, which is overexpressed in the cytoplasm and occurs on the surface of cancer cells. We investigated the therapeutic potential of aptamers in hepatocellular carcinoma (HCC) by evaluating anti-tumor effects and confirming the affinity and specificity of AS1411- and modified AS1411-aptamers in HCC cells. Cell growth was assessed using the MTS assay, and cell death signaling was explored by immunoblot analysis. Fluorescence-activated cell sorting was performed to evaluate the affinity and specificity of AS1411-aptamers in SNU-761 HCC cells. We investigated the *in vivo* effects of the AS1411-aptamer using BALB/c nude mice in a subcutaneous xenograft model with SNU-761 cells. Treatment with a modified AS1411-aptamer significantly decreased *in vitro* (under normoxic [*P* = 0.035] and hypoxic [*P* = 0.018] conditions) and *in vivo* (under normoxic conditions, *P* = 0.041) HCC cell proliferation compared to control aptamers. AS1411- and control aptamers failed to control HCC cell proliferation. However, AS1411- and the modified AS1411-aptamer did not induce caspase activation. Decrease in cell growth by AS1411 or modified AS1411 was not prevented by caspase or necrosis inhibitors. In a microarray, AS1411 significantly enhanced galectin-14 expression. Suppression of HCC cell proliferation by the modified AS1411-aptamer was attenuated by galectin-14 siRNA transfection. Modified AS1411-aptamer suppressed HCC cell growth *in vitro* and *in vivo* by up-regulating galectin-14 expressions. Modified AS1411-aptamers may have therapeutic potential as a novel targeted therapy for HCC.

## Introduction

Hepatocellular carcinoma is complex, with a heterogeneous tumor biology [1]. Several genomic alterations, such as Ras/extracellular signal-regulated kinases, epidermal growth factor receptor (EGFR), phosphoinositol 3-kinase/mammalian target of rapamycin (mTOR), hepatocyte growth factor/mesenchymal-epithelial transition factor, Wnt, and Hedgehog signaling, limit the therapeutic efficacy of systemic molecular targeted chemotherapy, including sorafenib [2–4]. Therefore, targeted treatment with high affinity to specific receptors on HCC cells is essential [5]. Nucleic acid ligands, or aptamers, are single-stranded oligonucleotides with a unique molecular conformation that have recently been proposed as new targeted therapies in oncology [6]. Aptamers within drug-encapsulated polymer particles have cell-specific anti-tumor effects. Combining targeted-delivery with controlled drug release leads to cytotoxicity in targeted tumor cells and spares surrounding noncancerous cells from toxicity, which is an ideal strategy for cancer treatment.

AS1411, previously known as AGRO100 or ACT-GRO-777, is a guanosine (G)-rich oligodeoxynucleotide with high affinity for nucleolin [7]. AS1411 is the first deoxyribonucleic acid (DNA) aptamer that has shown anti-proliferative activity against cancer cells, including acute myelogenous leukemia in phase I and II clinical trials [8]. Nucleolin is highly expressed at the surface of actively dividing HCC cells [9], and is reported to be correlated with biologically aggressive HCC progression and poor prognosis [10]. Chemical modification of 5-(N-benzylcarboxyamide)-2’-deoxyuridine(5-BzdU) in the AS1411-aptamer, which binds to nucleolin in cancer cells, was recently shown to increase binding and targeting affinity to cancer cells [11]. In this study, we evaluated the targeting affinity and anti-tumor efficacy of AS1411-and modified AS1411-aptamers on hepatoma cells and the mechanisms involved.

## Materials and Methods

### Aptamer DNA

The AS1411-aptamer (5′-GGTGGTGGTGGTTGTGGTGGTGGTGG-3′), control AS1411-aptamer (5'-CTTCTTCTTCTTTCTCTTCTTCTTCT-3'), and random control aptamer (5'-TGAGGCCGACATTGTTTAGAATCACC-3') were purchased from Bioneer (Daejeon, Republic of Korea) [7]. Control AS1411-aptamer was made by changing guanine to cytosine in guanine-quartet which forms a unique three-dimensional structure of AS1411-aptamer. Control AS1411-aptamer does not have a unique three-dimensional structure. A modified AS1411-aptamer (5’-GGTGGTGGTGGZZGTGGTGGTGGTGG-3', Z = 5-(N-naphthylcarboxyamide)-2'-deoxyuridine) was provided by ST Pharm (Seoul, Republic of Korea) [11].

### Cell line and Cell Culture

In this study, three human HCC cell lines were used: SNU-761, a poorly differentiated-HCC cell line [12]; Huh-7, a well-differentiated HCC cell line [13]; and SNU-3058, a hypovascular HCC cell line which was deposited to the Korea Cell Line Bank. Cells were grown in Dulbecco’s Modified Eagle Medium (DMEM) supplemented with 10% fetal bovine serum (FBS), 100,000 U/L penicillin, and 100 mg/L streptomycin with or without 100 nmol/L insulin. In all experiments, cells were starved overnight to avoid confounders related to serum-induced signaling. Cells were incubated under either normoxic (20% O_2_ and 5% CO_2_ at 37°C) or hypoxic conditions (1% O_2,_ 5% CO_2_, and 94% N_2_ at 37°C).

### Fluorescence Microscopy

To stain the plasma membrane and cytoplasmic nucleolin, cells were fixed for 20 min at room temperature in PBS (140 mmol/L NaCl, 2.7 mmol/L KCl, 10 mmol/L Na_2_HPO_4_, 1.8 mmol/L KH_2_PO_4_, pH 7.4) containing 4% paraformaldehyde. Nonspecific binding of antibody was blocked with 3% bovine serum albumin (BSA) and 1% goat serum in PBS (blocking buffer) for 1 hour at room temperature. Cells were incubated overnight at 4°C with primary anti-nucleolin antibody (Santa Cruz Biotechnology, clone MS-3; 1:100 dilution in blocking buffer), washed in PBS, and incubated with secondary FITC-conjugated goat anti-mouse IgG (diluted 1:100 in blocking buffer) for 1 h at room temperature. Nucleic acids were stained using the binding dye 4,6-diamidino-2-phenylindole dihydrochloride (DAPI). The cells were washed thrice in PBS and observed under a Carl Zeiss LSM5 Pascal confocal microscope.

### Flow cytometry

The specific binding affinities of the AS1411-aptamer or modified AS1411-aptamer were assessed by fluorescence-activated cell sorting (Becton Dickson, FACScan^®^, Mansfield, MA, USA). Three human HCC cell lines were used for fluorescence-activated cell sorting: SNU-761, a poorly differentiated HCC cell line; Huh-7, a well-differentiated HCC cell line [13]; and SNU3058, a hypovascular HCC cell line that was deposited into the Korea Cell Line Bank (KCLB). Binding affinities of the aptamers were determined by incubating cells (5×10^5^) on ice for 50 min in the dark with varying concentrations of Cy-3-labeled aptamer in a 500-μL volume of binding buffer containing 20% FBS. Cells were then washed twice with 0.7 mL of the binding buffer with 0.1% sodium azide, suspended in 0.4 mL of binding buffer with 0.1% sodium azide, and subjected to flow cytometric analysis within 30 min. The mean fluorescence intensity of the target cells labeled with aptamers was used to calculate specific binding by subtracting the mean fluorescence intensity of nonspecific binding from unselected DNA libraries. Equilibrium dissociation constants (K_d_) of the aptamer–cell interactions were obtained by fitting the dependence of fluorescence intensity of specific binding on aptamer concentrations to the equation Y = B_max_ X/(K_d_ + X). All binding assay experiments were repeated three times.

### Cell Proliferation Analysis (MTS Assay)

Cell proliferation was measured based on cellular conversion of the colorimetric reagent 3,4-(5-dimethylthiazol-2-yl)-5-(3-carboxymethoxyphenyl)-2-(4-sulfophenyl)-2H-tetrazolium salt (MTS) into soluble formazan by a dehydrogenase enzyme found in metabolically proliferating cells (CellTiter 96 Aqueous One Solution cell proliferation assay; Promega, Madison, WI). Following each treatment, 20 μL of dye solution was added into each well in a 96-well plate and incubated for 2 hours. Absorbance was recorded at a wavelength of 490 nm using an enzyme-linked immunosorbent assay (ELISA) plate reader (Molecular Devices, Sunnyvale, CA, USA).

### Cell cycle analysis

Flow cytometry cell cycle analysis using propidium iodide DNA staining was performed. Cells were harvested in the appropriate manner, washed in PBS, and fixed in cold 70% ethanol. To ensure fixation of all cells and minimize clumping, drop wise was added to the pellet while vortexing. Then, cells were fixed for 30 min at 4°C, washed two times in PBS, and spinned at 850 g in a centrifuge. Cells were treated with ribonuclease, and were added 50 μl of a 100 μg/ml sock of RNase. Forward scatter and side scatter were measured to identify single cells.

### Immunoblot Analysis

Cells were lysed for 20 min on ice with lysis buffer and centrifuged at 14,000 g for 10 min at 4°C. Samples were resolved by sodium dodecyl sulfate polyacrylamide gel electrophoresis (SDS–PAGE), transferred to nitrocellulose membranes, blotted with appropriate primary antibodies at a dilution of 1:1000, and treated with peroxidase-conjugated secondary antibodies (Biosource International, Camarillo, CA, USA). Bound antibodies were visualized using a chemiluminescent substrate (ECL; Amersham, Arlington Heights, IL, USA) and exposed to Kodak X-OMAT film (Kodak, New Haven, CT, USA). Primary antibodies included rabbit anti-phospho-ERK1/2 mitogen-activated protein kinase (MAPK), anti-phospho-Akt, anti-caspase 9 and anti-caspase 7 (cleaved) from Cell Signaling Technology (Danvers, MA, USA), rabbit anti-caspase 8 from BD Biosciences (San Jose, CA, USA), and mouse anti-vimentin from BioGenex (Dublin, CA, USA). Goat anti-actin antibody was obtained from Santa Cruz Biotechnology Inc. (Santa Cruz, CA, USA).

### Real Time-Polymerase Chain Reaction (PCR)

Total ribonucleic acids (RNAs) were extracted from SNU-761 cells using Trizol Reagent (Invitrogen, Carlsbad, CA, USA). Complementary deoxyribonucleic acid (cDNA) templates were prepared using oligo(dT) random primers and Moloney murine leukemia virus (MoMLV) reverse transcriptase. After the reverse transcription reaction, the cDNA template was amplified by PCR using Taq polymerase (Invitrogen). Galectin-14 was quantitated by real-time PCR (LightCycler; Roche Molecular Biochemicals, Mannheim, Germany) using SYBR green as the fluorophore (Molecular Probes, Eugene, OR, USA). After electrophoresis in 1% agarose gel, the portion of gel containing the expected galectin-14 PCR product was excised, and the product was eluted into Tris-HCl using a DNA elution kit (Qiagen, Valencia, CA, USA). Primers were as follows: forward: 5'-ATGTCCCTGACCCACAG-3' and reverse: 5'-TCAATCGCTGATAAGCACT-3'. Glyceraldehyde-3-phosphate dehydrogenase (GAPDH) gene expression was used as a control. Galectin-14mRNA expression levels were calculated as the relative intensity of the PCR product bands compared with intensity from the GAPDH gene using the 2–^△△Ct^ method. All PCR experiments were performed in triplicate.

### NF-κB activity assay

Cells were plated at 5×10^4^ cells/well in 1 mL of media in six-well plates and treated with or without aptamers. Nuclear fractions were extracted, and NF-κB concentrations (p50/p65) were determined using Trans Factor NF-κB p50/p65 chemiluminescent kits (Clontech, Mountain View, CA, USA).

### cDNA microarray analysis

To compare relative gene expression profiles in SNU-761 cells with AS1411-aptamer treatment, total RNA from SNU-761 cells treated with AS1411-aptamer or control were extracted and purified. Microarray analysis was performed according to the Macrogen Rat Bead Chip technical manual (Macrogen, Seoul, Korea) using the Illumina RatRef-12 Expression Bead Chip (Illumina, Inc., San Diego, CA, USA). Biotinylated cRNAs were prepared from 0.55 μg quantities of total RNA using the Illumina Total Prep RNA Amplification Kit (Ambion, Austin, TX, USA). Following fragmentation, cRNA was hybridized to the Illumina RatRef-12 Expression Bead Chip in 0.75 μg quantities using protocols provided by the manufacturer. Arrays were scanned using the Illumina Bead Array Reader Confocal Scanner. Array data export processing and analysis were performed using Illumina Bead Studio v3.1.3 (Gene Expression Module v3.3.8).

### In vivo subcutaneous xenograft model

Briefly, SNU-761 cells (5×10^7^ cells/mouse) were subcutaneously transplanted into the flanks of BALB/c nude mice. Tumor volume was measured using a vernier caliper and calculated as [length × (width)^2^]/2. When the tumor volume reached a size of about 200 mm^3^, mice were injected with AS1411- (n = 6), modified AS1411- (n = 6) or control aptamers (n = 6). Peritumoral injections (10 mg/kg in 100 μL DPBS buffer) were performed six times daily for the first three days and then every other day for an additional three treatments.

Mice were housed in ventilated cages, maximum 5 per cage, in a 12-hour light/dark cycle. Animals received sterile food and water and were handled aseptically. Animals were monitored at least twice daily with health monitor forms. None of the animals involved in this study showed sign of illness or died prior to the experimental endpoint. Any animal deemed to be at humane endpoint was euthanized. Criteria used for humane endpoint for the following experiments included one or more of the following: loss of >20% body weight, labored breathing, lack of response to stimulus and lethargic animal. We did not perform surgical procedures as part of this study.

### Statistical analysis

Statistical analyses were performed using PASW version 22.0 (IBM, Chicago, IL, USA). All experimental results were obtained from three independent experiments using cells from three separate isolations and are presented as the mean ± standard deviation (SD). For comparisons between groups, data were analyzed by the Mann–Whitney U test or one-way ANOVA. For all tests, *P* <0.05 was regarded as statistically significant.

### Ethics Statement

Ethics approval from the ethics committee at Seoul National University Hospital was obtained. We carried out this study in strict accordance with the recommendations in the Guide for the Care and Use of Laboratory Animals of the National Institutes of Health. *In vivo* study protocol was approved by the Institutional Animal Care and Use Committee (IACUC No. 15-0003-C2A0) of Seoul National University Hospital.

## Results

### Nucleolin expression in HCC cells

**Fig 1:**
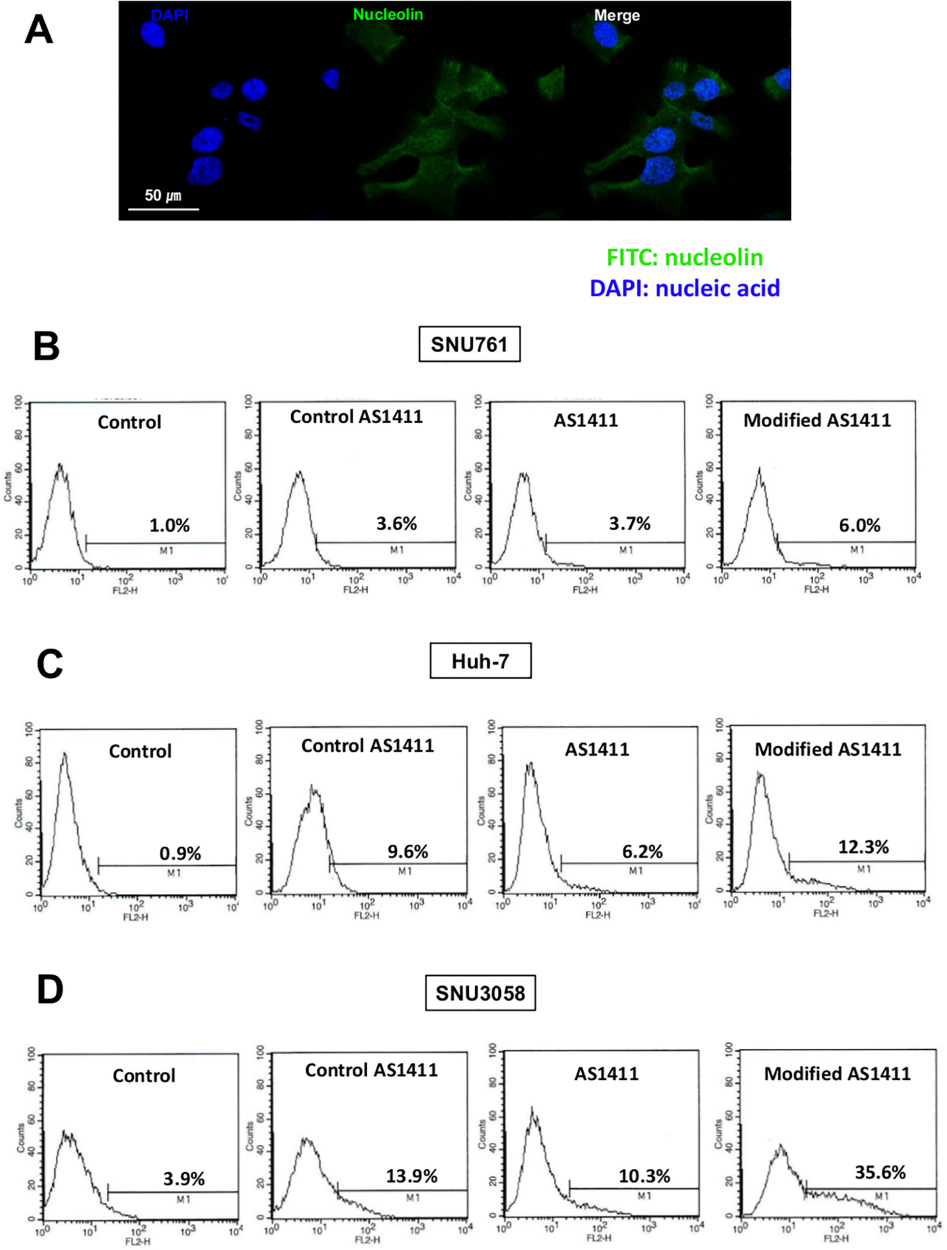
Nucleolin expression in HCC cells. (A) Nucleolin (green fluorescence) was present on the surface of SNU-761 cells based on fluorescence microscopy. (B−D) Flow cytometry analysis of cells immunostained with Cy-3 (1:400, 100 nM) showed that AS1411- and modified AS1411-aptamers had relatively higher affinity to SNU-761 (B), Huh-7 (C) and SNU3058 cells (D) compared to control.

Because AS1411-aptamer targets nucleolin on the cell surface, we evaluated nucleolin expression on the cell surface in SNU-761 HCC cells. We conjugated FITC to an anti-nucleolin antibody and stained SNU-761 cells. Nuclei were stained with DAPI. Fig 1A shows that nucleolin (green fluorescence) is present on the surface of SNU-761 cells.

### AS1411- and modified AS1411-aptamer binding affinity for HCC cells

We determined the binding affinity of AS1411- and modified AS1411-aptamers for HCC cell lines, including SNU-761, Huh-7, and SNU3058 cells. Flow cytometry analysis of immunostained cells using Cy-3 (1:400, 100 nM) showed that both AS1411- and modified AS1411-aptamers had a relatively high affinity for SNU-761 cells (3.7% and 6.0%, respectively) compared to control and control AS1411-aptamers (Fig 1B). AS1411- and modified AS1411-aptamers also exhibited higher affinity for Huh-7 cells (Fig 1C; 6.2% and 12.3%, respectively) and SNU3058 cells (Fig 1D; 10.3% and 35.6%, respectively) compared to control.

### In vitro and in vivo anti-tumor effects of AS1411- and modified AS1411-aptamers on SNU-761 cells

**Fig 2:**
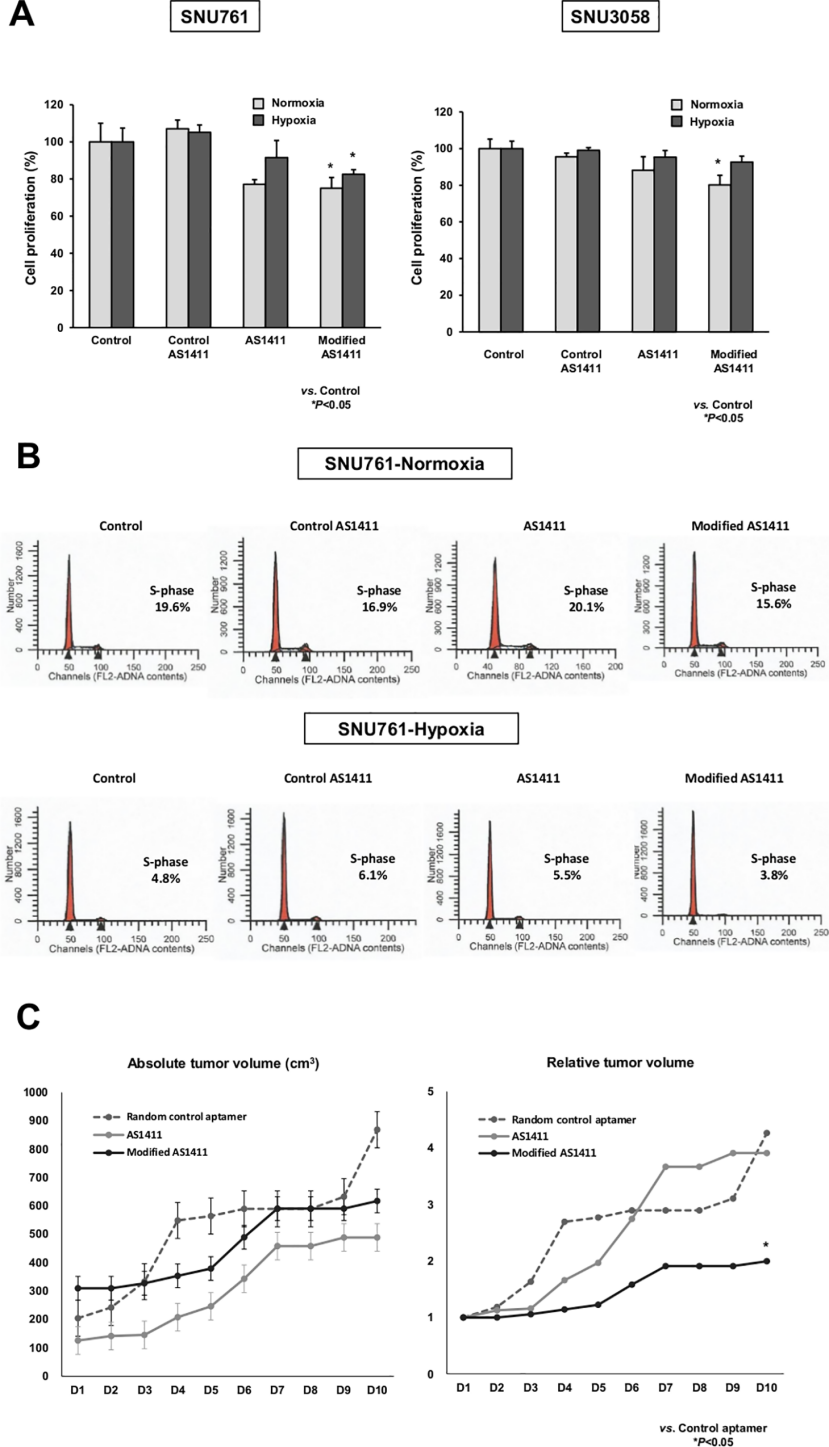
Anti-tumor effects of modified AS1411-aptamers on SNU-761 cells. (A) Significant cytotoxic effects on SNU-761 cells were noted with modified AS1411-aptamer treatment under both normoxic and hypoxic conditions. (B) Cell cycle analysis revealed that modified AS1411-aptamer significantly decreased the proportion of S-phase cells as compared to control AS1411-aptamer, especially under hypoxic conditions (*P* = 0.009). The experiment was repeated three times. (C) In an *in vivo* xenograft model, modified AS1411-aptamers significantly suppressed HCC tumor volume by 53.2% compared to control aptamers at day 10 (*P<0.05).

We evaluated the potential anti-proliferative effects of AS1411- (10 μM) and modified AS1411-aptamers (10 μM) on SNU-761 cells *in vitro*. SNU-761 cells were grown under normoxic or hypoxic conditions. Cell viability was assessed using the MTS assay after 48 hr of aptamer treatment. Control AS1411-aptamer did not significantly suppress HCC cell proliferation. AS1411-aptamer decreased HCC cell proliferation under both normoxic and hypoxic conditions, although not significantly. Significant cytotoxic effects on SNU-761 cells were noted with modified AS1411-aptamer under normoxic (*P* = 0.035) and hypoxic (*P* = 0.018) conditions (Fig 2A). Modified AS1411-aptamer also showed significant cytotoxic effects on SNU-3058 cells under normoxic conditions (*P* = 0.01). Moreover, cell cycle analysis revealed that modified AS1411-aptamer significantly decreased the proportion of S-phase cells as compared to control AS1411-aptamer, especially under hypoxic condition (Fig 2B; *P* = 0.009).

Anti-tumor effects of modified AS1411-aptamer were examined using an *in vivo* xenograft model. When the tumor volume reached a size of approximately 200 mm^3^ after inoculation of 5×10^7^ cells into the flank, peritumoral subcutaneous injection of AS1411-, modified AS1411- or random control aptamers was performed. Day 1 (D1) represents the first day of peritumoral aptamer injection. After six subcutaneous peritumoral injections, the modified AS1411-aptamer significantly suppressed HCC cell proliferation compared to random control aptamer at day 10 (D10) (Fig 2C). When initial tumor volume (D1) was standardized to 1, the relative tumor volume of the random control aptamer group at D10 was 4.26, while that of the modified AS1411-aptamer group was 2.0. The modified AS1411-aptamers decreased the tumor volume by 53.2% compared to control aptamers at D10 (*P* = 0.041). The relative tumor volume of the AS1411-aptamer group at D10 was 3.91. No significant growth inhibition was observed in mice treated with the AS1411-aptamer compared to a random control aptamer at D10 (*P* = 0.233).

### The anti-tumor mechanism of AS1411- or modified AS1411-aptamersin SNU-761 cells: galectin-14

**Fig 3:**
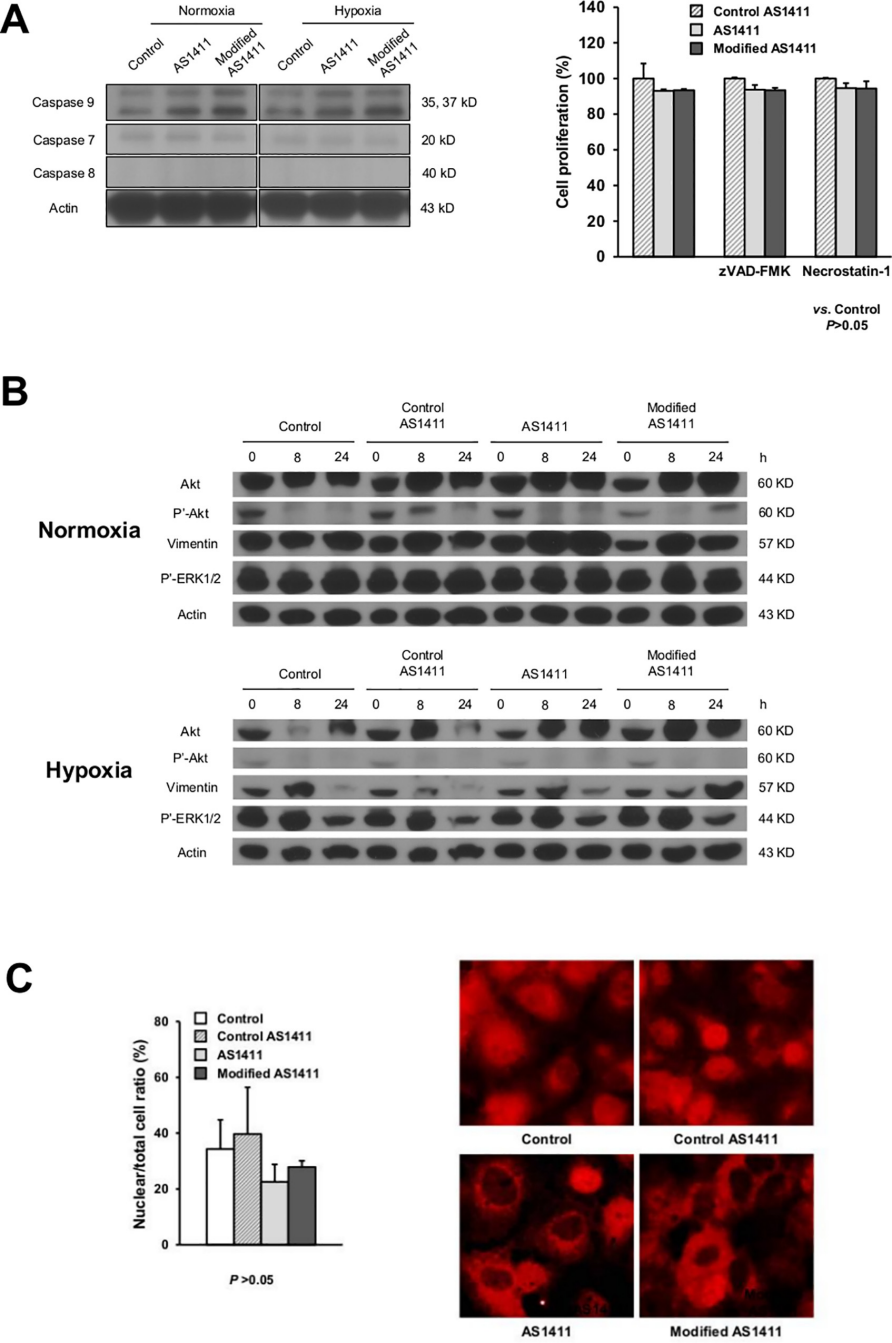
The mechanism of AS1411-or modified AS1411-aptamersregarding anti-tumor effects in SNU-761 cells. (A) No significant induction of SNU-761 cell apoptosis was noted with AS1411- or modified AS1411-aptamers. Immunoblot analysis was performed using anti-caspase 9, anti-caspase 8, anti-caspase 7 and anti-actin antibodies. (B) No significant changes in phosphorylated Akt (P’-Akt) and phosphorylated ERK1/2 (P’-ERK1/2) expression were noted following AS1411- or modified AS1411-aptamer treatment. (C) The percentage of nuclei stained with NK-κB (p65) was not significantly decreased followingAS1411- or modified AS1411-aptamer treatment (all *P*>0.05).

AS1411- and modified AS1411-aptamers exhibited *in vitro* and *in vivo* anti-tumor effects in SNU-761 cells. However, only the modified AS1411-aptamer showed statistical significance. We did not observe significant induction of SNU-761 cell apoptosis with AS1411- or modified AS1411-aptamers. As shown in Fig 3A, caspase 9, 7, and 8 expressions did not change significantly with AS1411- or modified AS1411-aptamer treatment compared to control-aptamers. The pan-caspase inhibitor zVAD-FMK did not significantly change AS1411- or modified AS1411-treated SNU-761 cell viability, nor did necrostatin-1, a potent selective inhibitor of necroptosis.

To find the main mechanism of anti-tumor activity, we evaluated whether the PI3K/Akt or ERK1/2-MAPK pathways were activated by AS1411- or modified AS1411-aptamers, as these are key survival signaling pathways in HCC cells [14]. However, no significant changes in phosphorylated Akt and phosphorylated ERK1/2 expression were noted following AS1411- or modified AS1411-aptamer treatment. Vimentin expression, which is a widely recognized epithelial-mesenchymal transition (EMT) marker, slightly decreased after modified AS1411-aptamer treatment under normoxic conditions (Fig 3B).

Mechanistic investigations have showed that nucleolin directly activates NF-κB signaling [15]. Therefore, we assessed the inhibitory effects of the AS1411-aptamer on NK-κB. NK-κB activity and nuclear translocation were measured using immunofluorescent microscopy. The percentage of nuclei stained with NK-κB (p65) was not significantly decreased by AS1411- or modified AS1411-aptamer treatment (Fig 3C; *P =* 0.185 and *P* = 0.397, respectively).

**Fig 4:**
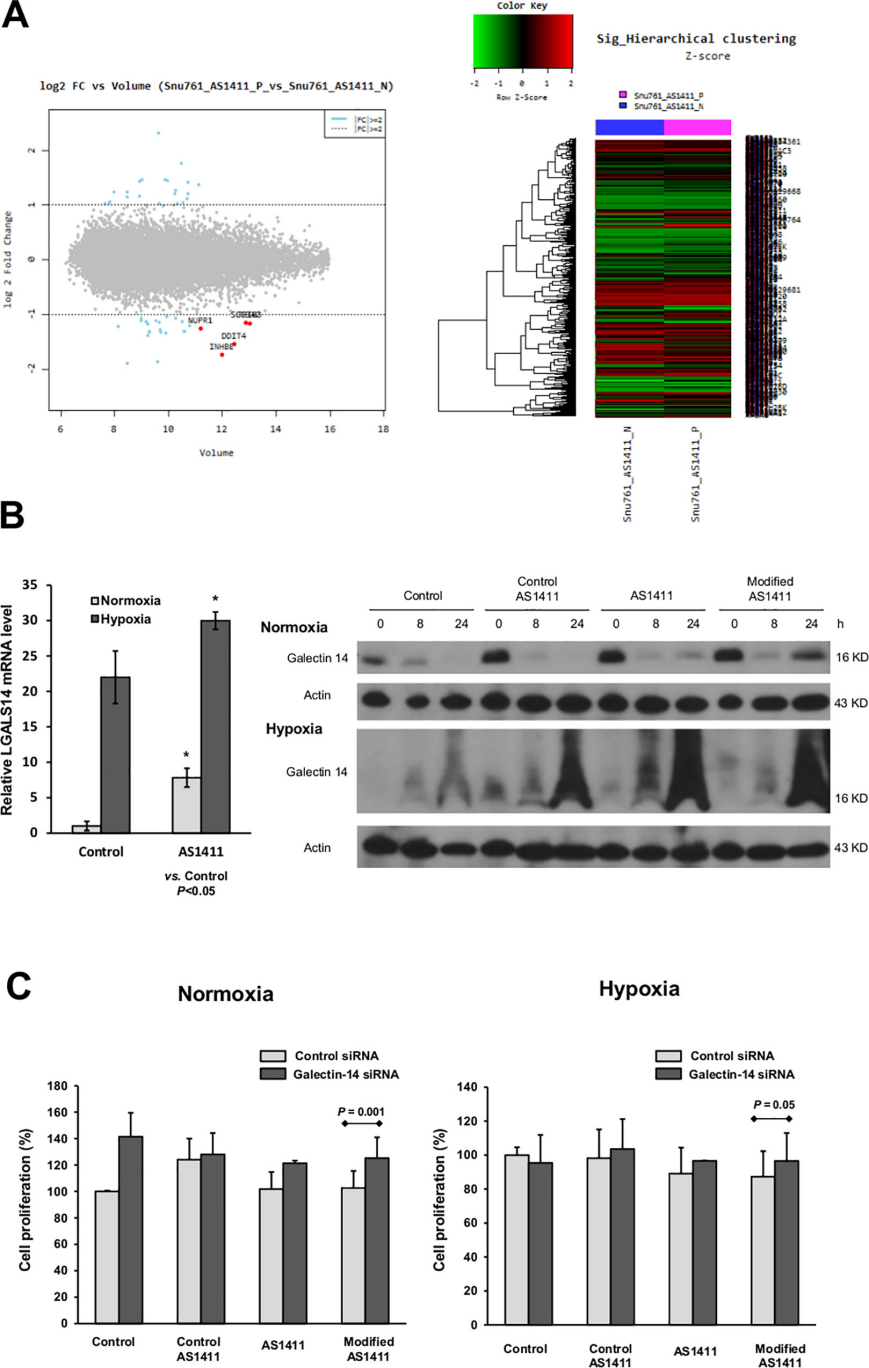
AS1411-aptamer suppresses hepatocellular carcinoma by up-regulating galectin-14. (A) DNA microarray results (volume plot of expression level, hierarchical clustering analysis) (B) Induction of LGALS-14 by the AS1411-aptamer was confirmed by RT-PCR under normoxic (20% O_2_ and 5% CO_2_ at 37°C) and hypoxic conditions (1% O_2_, 5% CO_2_, and 94% N_2_ at 37°C). Galectin-14 protein expression was also enhanced by both AS1411- and modified AS1411-aptamers based on immunoblot analysis. (C) Decreased galectin-14 expression by siRNA enhanced HCC proliferation compared to control siRNA, particularly with treatment of the modified AS1411-aptamer.

We examined molecules that were significantly modulated in SNU-761 cells by AS1411-aptamers using cDNA microarray analysis (Fig 4A). According to analysis, LGALS14 gene expression, which codes galectin-14, was significantly increased by the AS1411-aptamer under normoxic conditions, with a 4.98-fold change (*P*<0.0001). Induction of LGALS14 by the AS1411-aptamer was confirmed by RT-PCR under both normoxic and hypoxic conditions. Moreover, galectin-14 protein expression was also enhanced by both AS1411- and modified AS1411-aptamers on immunoblot analysis (Fig 4B).

To confirm the functional role of galectin-14 in HCC proliferation, we performed MTS assay with knockdown of galectin-14 by small interfering RNA (siRNA) transfection under both normoxic and hypoxic conditions. As shown in Fig 4C, decreased galectin-14 expression with siRNA transfection enhanced HCC proliferation compared to control siRNA, particularly under treatment with the modified AS1411-aptamer. Suppression of HCC cell proliferation by the modified AS1411-aptamer was attenuated by galectin-14 siRNA transfection under both normoxic (*P* = 0.001) and hypoxic conditions (*P* = 0.05).

## Discussion

We confirmed *in vitro* and *in vivo* anti-tumor effects of modified AS1411-aptamers on HCC cells. We also found that modified AS1411-aptamers suppressed HCC cell proliferation by modulating galectin-14 expression. Modified AS1411-aptamers were developed to improve the target affinity of aptamers, with affinities 2.5-fold higher than AS1411-aptamers [11]. Chemical modification of the pyrimidine base backbone of AS1411 nucleotides increases the binding affinity of aptamers. Herein, we found that the AS1411-aptamer did not induce apoptosis or necrosis in SNU-761 cells. The AS1411-aptamer did not modulate survival pathways, including PI3K/Akt and ERK1/2 MAPK. However, the AS1411-aptamer activated up-regulation of the protein galectin-14, which is a soluble β-galactoside-binding animal lectin.

Aptamers are oligonucleotide ligands that act as biological antibodies with high affinity binding to molecular targets and are preferred for cancer diagnosis and treatment. They are rapidly produced, highly stable, efficient and inexpensive. Aptamers are small in size (8−15 kDa) and are nonimmunogenic and nontoxic *in vivo* [16]. Some aptamers have already reached clinical stage testing, including macugen (pegaptanib sodium), which is a RNA aptamer against vascular endothelial growth factor used for neovascular age-related macular degeneration [17].

To improve the bioavailability of aptamers, modifications have been developed, including chemical modification of the backbone or side chain. Conjugation of aptamers with polyethylene glycol (PEG) is another strategy to overcome problems with rapid renal filtration. PEG modification, or PEGylation, increases the molecular weight of the aptamer beyond the renal filtration threshold of 40 kDa [18]. These aptamer modification strategies result in superior *in vivo* bioavailability and higher aptamer affinity. Chemical modification only enhances the binding and targeting affinity to targets of interest. Modified AS1411-aptamers also target nucleolin with same molecular mechanism of AS1411-aptamers.

Galectins are expressed on cells in the immune system and regulate immune cell responses and homeostasis [19]. To date, 14 members of the family have been characterized in mammals with essential functions including development, differentiation, growth regulation, apoptosis and tumor metastasis [20]. It has been reported that galectin acts as a potential immune-modulating agent that provides inhibitory or stimulatory signals to control immune cell response. Recently, it was reported that galectin-9 suppresses HCC growth *in vitro* and *in vivo*, suggesting it as a candidate for HCC chemotherapy [21]. The anti-tumor effects of galectin-14 against HCC have not yet been evaluated. In this study, we report a novel role of galectin-14 as a HCC suppressor. With the AS1411-aptamer, we were able to modulate galectin-14, which is a novel target for immunomodulation.

Different pattern of galectin-14 activation was observed between hypoxic and normoxic conditions. Due to a significant difference in molecular mechanism between hypoxic and normoxic conditions, the galectin-14 expression at early time (0 h) was not activated due to hypoxia-induced HCC cell proliferation. While, galectin-14 was activated at 24 h due to the effect of AS1411- or modified AS1411-aptamers under hypoxic conditions. Moreover, modified AS1411-aptamer significantly decreased the proportion of S-phase cells as compared to control AS1411-aptamer, especially under hypoxic conditions. Despite hypoxia induces survival in HCC by activating a variety of growth factor signals [22], modified AS1411-aptamer effectively arrested cell cycle under hypoxic conditions.

The AS1411-aptamer is currently in phase II clinical trials for patients with renal cell carcinoma and acute myeloid leukemia [8, 23]. Previous studies have shown that AS1411-aptamers bind to nucleolin on the plasma membrane and induce tumor cell apoptosis. In this study, the AS1411-aptamer had limited anti-proliferative effects in HCC cells. However, modification of the AS1411-aptamer significantly improved inhibitory effects on HCC cell proliferation *in vitro* and *in vivo*. Aptamers are identified by a process called systematic evolution of ligands by exponential enrichment (SELEX), which is based on a high affinity for targets [24]. Using 2’-amino or -fluoro pyrimidines, chemical modification of the ribose backbone in aptamer nucleotides has been performed to improve transfer and binding to the target. In initial findings, chemical modification frequently altered the structure of aptamers, resulting in a loss of properties [25]. Recently, a successful post-SELEX chemical modification of thymidines at the central or 3’ terminal region of AS1411 with 5-BzdU was reported, resulting in a more stable G-quadruplex structure via hydrophobic cavities and enhanced binding affinity of to cancer cells [11].

Herein, we observed anti-tumor effects of AS1411-aptamers and modified AS1411-aptamers targeting nucleolin, which is highly expressed in HCC cells. Oncofetal proteins, including alpha-fetoprotein and glypican-3, are overexpressed in HCC cells [26]. Therefore, targeting oncofetal proteins with specific aptamers might improve the effectiveness of HCC treatment strategies with fewer adverse events.

## Conclusion

In this study, we report the superior anti-proliferative effects of modified AS1411-aptamers on HCC cells compared to control- and AS1411-aptamers. Post-SELEX chemical modification produces stable and/or high affinity aptamers with electrostatic, hydrophobic interactions between nucleic acids and their targets. Post-SELEX modification does not increase time or labor as part of the SELEX procedure. Therefore, chemical modification might be directly applied to alter AS1411-aptamers with increasing binding affinity for nucleolin. The results of this study suggest the potential for chemically modified AS1411-aptamers as a HCC treatment modality.

## Abbreviations

HCC: hepatocellular carcinoma
DNA: deoxyribonucleic acid
RNA: ribonucleic acid
cDNA: complementary deoxyribonucleic acid
MAPK: mitogen-activated protein kinase
siRNA: small interfering RNA
WME: William's medium E
MTS: 3,4-(5-Dimethylthiazol-2-yl)-5-(3-carboxymethoxyphenyl)-2-(4-sulfophenyl)-2H-tetrazolium salt
ELISA: enzyme-linked immunosorbent assay
PCR: polymerase chain reaction
DMEM: Dulbecco’s Modified Eagle Medium
FBS: fetal bovine serum
